# Unconventional Rocky Mountain Spotted Fever Presentation From Kentucky: A Compelling Case Report and Literature Review

**DOI:** 10.7759/cureus.48558

**Published:** 2023-11-09

**Authors:** Akbar Hussain, Christopher Gray, Stanley Marlowe, Nazneen Ahmed, Fares Khater, Avinash Vangara

**Affiliations:** 1 Internal Medicine, Appalachian Regional Healthcare, Harlan, USA; 2 Internal Medicine, Lincoln Memorial University DeBusk College of Osteopathic Medicine, Harrogate, USA

**Keywords:** hyponatremia, tick-borne diseases, rickettsia rickettsii, doxycycline, rocky mountain spotted fever (rmsf)

## Abstract

Rocky Mountain spotted fever (RMSF) is a potentially lethal tick-borne disease caused by Rickettsia rickettsii, known for its tropism for vascular endothelial cells. Its classic symptoms include fever, headaches, and a rash, but atypical presentations can challenge diagnosis. We present the case of a 71-year-old male with fever, weakness, and hiccups, evolving into confusion. Laboratory findings showed severe hyponatremia, leukocytosis, and abnormal blood parameters. Initial management addressed sepsis and hyponatremia, leading to symptom improvement. Later, a fever of 106.5°F prompted ICU transfer, broad-spectrum antibiotics, and testing for tick-borne diseases. The patient reported tick exposure and received prophylactic doxycycline. Follow-up confirmed the RMSF diagnosis based on serological testing and clinical symptoms. This case highlights the diagnostic challenges posed by atypical RMSF presentations and underscores the importance of early detection and treatment to prevent complications.

## Introduction

Rocky Mountain spotted fever (RMSF), a Rickettsial infection, can impact various organ systems within the human body. According to the Centers for Disease Control and Prevention (CDC), five states in the United States, namely Missouri, Tennessee, Oklahoma, Arkansas, and North Carolina, have a notable prevalence of RMSF, although cases have also been documented in other states [1]. RMSF typically occurs in the summer but can manifest throughout the year. In the eastern United States, the American dog tick (Dermacentor variabilis) is primarily responsible for RMSF transmission, whereas the Rocky Mountain wood tick (Dermacentor andersoni) carries it in the Rocky Mountains. In parts of the southwestern U.S., the brown dog tick (Rhipicephalus sanguineus) has also become a significant vector for the disease [2]. The intracellular bacteria with tropism to vascular endothelial cells, enable it to affect any organ, potentially leading to multisystem failure. RMSF initially manifests with vague symptoms like fever, headache, rash, muscle pain, and nausea, which can be easily confused with other medical conditions. Early in the disease course, laboratory results often fall within normal ranges, and it may take several weeks to obtain confirmatory diagnostic outcomes [3-5].

Early clinical assessment is of paramount importance in managing RMSF, as administering treatment within the initial five days of illness significantly diminishes disease severity and the likelihood of fatality [5,6]. The decision to initiate treatment hinges on clinical suspicion and failure to promptly administer appropriate therapy during the first five days of illness is associated with elevated mortality rates [4,7,8]. Both the American Academy of Pediatrics and the Centers for Disease Control and Prevention concur that doxycycline is the primary treatment choice for RMSF across all age groups [9,10]. We present a 71-year-old male with a history of bladder cancer and other medical conditions who presents with fevers, weakness, and persistent hiccups. Initial tests and treatments did not reveal a cause, but later, after possible tick and lice exposure, he tested positive for Rickettsia and was prescribed prophylactic doxycycline during follow-up with infectious disease.

## Case presentation

We present the case of a 71-year-old male who arrived at the emergency department with complaints of subjective fevers, generalized weakness with myalgia, and persistent hiccups over the last two days. Notably, upon arrival, the patient displayed increasing confusion and agitation. His medical history included bladder cancer in remission, hypertension, peripheral artery disease, a history of smoking, and moderate alcohol consumption.

**Figure 1:**
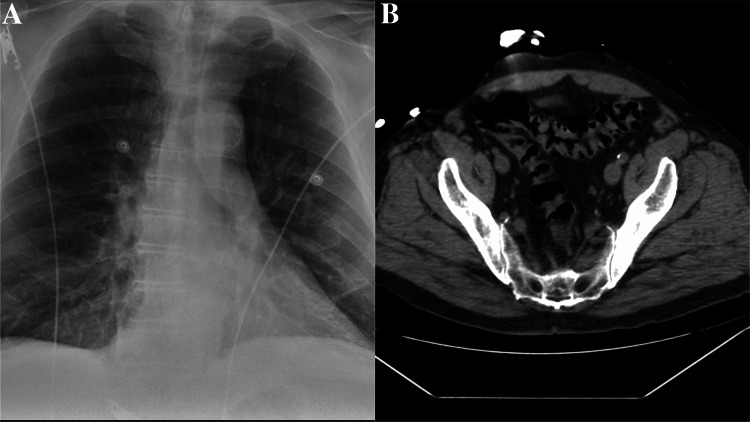
(A) Emphysematous changes in the chest x-ray and (B) diverticulosis revealed in abdominal CT.

During the physical examination, the patient exhibited tachycardia (heart rate: 110/min), tachypnea (respiratory rate: 20/min), elevated blood pressure (150/90 mmHg), and a high fever, peaking at 103.3°F. Additionally, he experienced intractable hiccups. Urinalysis revealed trace leukocyte esterase, 6-10 white blood cells, and trace bacteria. Initially, his sodium level was low (Na 124) but later improved with guideline-directed therapy, remaining at 127, as shown in Table 1. The patient also presented with elevated creatinine kinase (CK) levels, although the electrocardiogram did not reveal ischemic changes or findings suggestive of myocarditis. Chest x-ray results showed emphysematous changes, and a CT scan of the abdomen and pelvis indicated diverticulosis as shown in Figures 1A, 1B. Additionally, in our case, it is worth noting that this is associated with mildly elevated liver function tests (LFTs), which adds to the complexity of the clinical presentation as shown in Table 1.

**Table 1:** Patient lab values on admission

Parameter	Result	Normal Range
WBC (White Blood Cell)	6.49 x10^3/µL	4.23-9.07 x10^3/µL
RBC (Red Blood Cell)	4.50 x10^6/µL	4.63-6.08 x10^6/µL (L)
Platelet Count	144 x10^3/µL	163-337 x10^3/µL (L)
MPV (Mean Platelet Volume)	92fL	9.4-12.4 fL (L)
Neutrophil %	93.5%	34.0-67.9% (H)
Lymphocyte %	2.9%	21.8-53.1% (L)
Monocyte %	2.5%	5.3-12.2% (L)
Eosinophil %	1.1%	0.8-7.0% (L)
Basophil %	0.0%	0.2-1.2% (L)
Immature/Total Granulocyte Ratio	1.100%	0-0.429% (H)
Lactic Acid	3.8 mmol/L	0.4-2.0 mmol/L (H)
Total Bilirubin	0.40 mg/dL	0.20-1.00 mg/dL
AST (Aspartate Aminotransferase)	57 U/L	15-37 U/L (H)
ALT (Alanine Aminotransferase)	55 U/L	16-63 U/L
Alkaline Phosphatase	TOU/L	46-116 U/L
Total Creatine Kinase	442 U/L	39.00-308.00 U/L
Troponin	353 ng/L	4-76 ng/L

The patient's condition showed improvement following the initiation of the sepsis protocol for urinary tract infection or pneumonia, which included the administration of normal saline and IV ceftriaxone, as well as pending blood and urine cultures. Hyponatremia was closely monitored with intravenous normal saline and scheduled repeat BMPs to assess sodium levels. Fever management with Tylenol was effective, and the patient's altered mental status, attributed to hyponatremia and urosepsis, improved. Intractable hiccups were addressed through osteopathic manual manipulation and myofascial release. Hypertension management continued with bisoprolol, and thiazides were temporarily discontinued due to hyponatremia, with plans to resume once resolved. The patient was transferred from the intensive care unit (ICU) to the floor with a fever under control and improved urine output, and no new concerning symptoms were reported.

Later on, he developed a fever of 106.5 F. He was transferred to the intensive care unit for further management. Piperacillin tazobactam and vancomycin were added for broad-spectrum coverage in addition to obtaining blood cultures and a respiratory viral panel, which all returned negative. The patient was transferred to the floor after stabilizing his fever. Upon further history-taking to determine the source of a fever of unknown origin, the patient revealed a potential exposure to ticks and a cat suspected of having lice one week prior. Subsequently, the patient was tested for Bartonella, Borrelia burgdorferi, and Rickettsia. Despite experiencing intermittent fevers, the patient requested discharge due to an improvement in symptoms. As a precautionary measure given the exposure history, empirical doxycycline was prescribed. The patient was then scheduled for a follow-up with an infectious disease specialist.

During the follow-up, the patient, who initially presented with symptoms such as fever, headache, muscle pain, and fatigue but did not exhibit the typical RMSF rash, underwent further testing. The initial serology results were inconclusive. However, during the follow-up, the patient tested positive for Rickettsia with IgG and IgM antibody levels of 1.8 U/mL and 2.5 U/mL against RMSF, respectively. This atypical presentation raised concerns, and considering RMSF as part of the differential diagnosis was crucial. Empiric treatment with doxycycline was initiated, which played a pivotal role in preventing potential complications. Furthermore, it's noteworthy that repeat RMSF serology showed an IgG titer of 1:16 by EIA. Importantly, despite these findings, Ehrlichia (anaplasmosis) PCR testing returned negative results.

These collective findings ultimately confirm a diagnosis of RMSF, underscoring the importance of immediate medical attention and the need for appropriate antibiotic treatment to effectively manage the infection and mitigate potential complications. The patient's healthcare provider should be consulted for further evaluation and tailored treatment planning.

## Discussion

**Figure 2:**
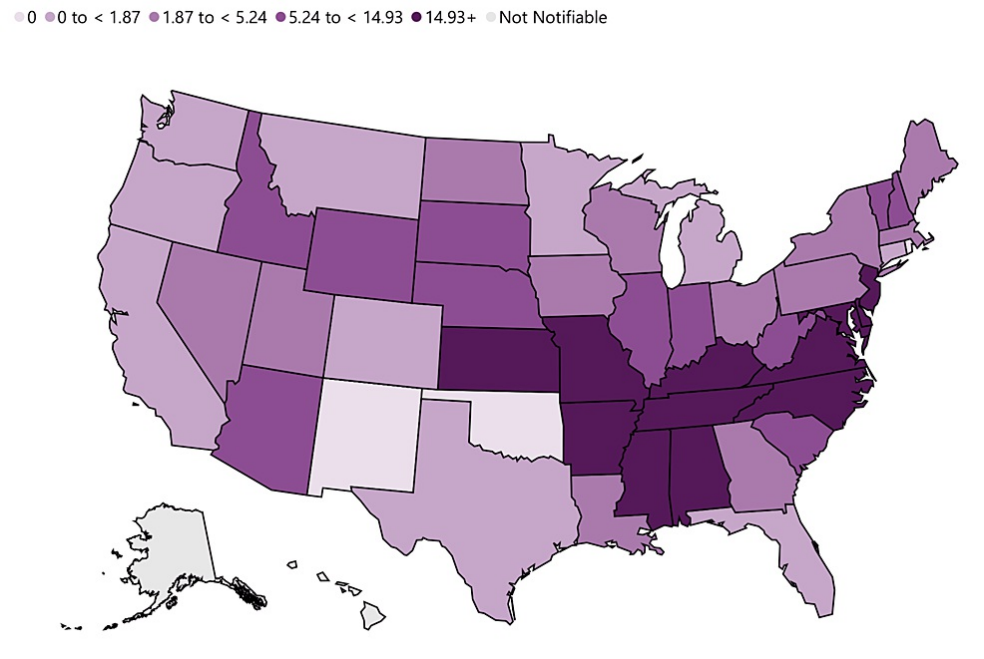
Annual incidence (per million population) of reported spotted fever Rickettsioisis – United States, 2019.

RMSF is an infectious disease resulting from a tick bite, impacting over 2,000 individuals annually in the United States. While it predominantly occurs between April and September, it can manifest throughout the warm months as shown in Figure 2 [1]. Initially identified in the Rocky Mountain states, RMSF can now be found across the nation, with a higher incidence in the southeastern and south-central regions. It is important to note that the disease is transmitted through tick bites and does not spread from person to person [11].

RMSF, a rapidly progressing and potentially lethal disease, underscores the urgency of early detection and treatment to avert fatalities. Diagnostic results are typically unavailable within the first five days, emphasizing the pivotal role of clinical judgment in prompt intervention. Although the classic trio of fever, rash, and tick bite is commonly relied upon for RMSF identification, our review underscores their inconsistency and unreliability as exclusive diagnostic criteria. Healthcare providers should consider these indicators while recognizing that the absence of tick exposure, fever, or rash does not eliminate the possibility of RMSF. While a history of a tick bite can assist in assessing risk, it may not always be evident in confirmed RMSF cases. Notably, we observed that 14% of patients did not report a fever during their illness. Although fever has traditionally been a diagnostic hallmark, its absence should not preclude clinical consideration, especially in endemic regions when patients otherwise exhibit compatible symptoms [6,12,13]. In our case, the patient initially presented with a fever of unknown origin, which later resolved. This shift in symptoms redirected our focus away from the primary suspicion of rickettsia infection, leading us to continue treating the patient for a fever of unknown origin.

Clustered RMSF cases, particularly when involving animals like dogs, can serve as crucial indicators of the disease's presence in the local tick population. In a review cluster involving dogs and humans, delayed recognition led to fatalities, highlighting the importance of awareness and collaboration between veterinary and human healthcare providers [14]. In our patient's case, an in-depth medical history uncovered a fever of unknown origin. The patient's disclosure of recent tick exposure and contact with a cat suspected of having “lice” one week earlier raised concerns about the possibility of a rickettsial disease.

RMSF diagnostics vary in effectiveness. The gold standard, the immunofluorescence assay (IFA), compares IgG titers between acute and convalescent samples but has limitations. IgG antibodies may take time to detect and can persist long after the infection or develop slowly, sometimes being missed [6]. IgM IFA's use has declined due to specificity issues. PCR on tissue samples is sensitive but affected by prior doxycycline use. Immunohistochemistry is effective but less accessible. Cultures are rarely used because R. rickettsii is an obligate intracellular organism that's hard to culture [6]. In our case, the RMSF diagnosis relied on clinical symptoms, exposure history, and positive serological tests for IgG and IgM antibodies. The combined findings confirmed RMSF, emphasizing the significance of comprehensive diagnostics and prompt treatment. Doxycycline is the recommended treatment for RMSF, significantly reducing morbidity and mortality when administered within the first five days of symptoms. Failure to consider RMSF in the initial diagnosis can lead to treatment delays [6-9]. The recent review reveals inadequate timely doxycycline or anti-rickettsial therapy, with a median initial treatment delay, especially pronounced in patients aged 18 [2]. Early doxycycline initiation is vital for reducing mortality, and recent data suggests improvements, emphasizing the need for ongoing healthcare provider education.

Atypical manifestations of RMSF, such as generalized neurologic complaints, myocarditis, or visual disturbances, are not well characterized in the literature. Healthcare providers should consider RMSF in the differential diagnosis of patients with these manifestations. Further documentation and characterization are needed to deepen clinical knowledge, prevent adverse outcomes, and enhance diagnosis. Chronic sequelae following RMSF are not fully understood, with neurologic deficits and necrosis of the skin or extremities being common. Lack of information about long-term consequences contributes to uncertainty for providers and patients after acute illnesses.

## Conclusions

This case report underscores the diagnostic challenges posed by atypical presentations of RMSF and emphasizes the critical importance of early detection and treatment to prevent severe complications. RMSF, caused by R. rickettsii and transmitted through tick bites, can manifest with a wide range of clinical symptoms, making diagnosis challenging, especially when the classic triad of fever, rash, and tick bite is absent. In this case, a 71-year-old patient initially presented with fever, weakness, and hiccups, which evolved into confusion, leading to diagnostic uncertainty. Timely management addressing sepsis and hyponatremia improved the patient's condition, but subsequent high fevers prompted an intensive care unit transfer and further investigation. The patient's history of tick exposure and positive serological tests confirmed the RMSF diagnosis, highlighting the significance of considering this disease in endemic regions. Early initiation of doxycycline is vital to reducing the morbidity and mortality associated with RMSF. This case also underscores the need for ongoing healthcare provider education on atypical RMSF presentations and the importance of comprehensive diagnostics.
